# Transcriptome analysis of injured muscle identifies new candidate genes for satellite cell growth and myofiber formation during early muscle regeneration

**DOI:** 10.5713/ab.24.0859

**Published:** 2025-08-12

**Authors:** Zhuning Yuan, Xian Tong, Xianyao Luo, Liping Pan, Hoi-ka Wu, Rong Xu, Ziyun Liang, Xunhe Huang, Delin Mo

**Affiliations:** 1 Conservation and Utilization Laboratory of Mountain Characteristic Resources in Guangdong Province, School of Life Science, Jiaying University, Meizhou, China; 2 State Key Laboratory of Biocontrol, School of Life Sciences, Sun Yat-Sen University, Guangzhou, China

**Keywords:** Cell Adhesion, Immune System, Myofiber Formation, Satellite Cell Growth, Transcriptome

## Abstract

**Objective:**

The self-repair capacity of skeletal muscle makes satellite cell activity and myofiber formation interesting. The major molecular networks of satellite cell activity have been extensively studied. However, the mechanism by which micro-environmental factors regulate satellite cell activity for early muscle regeneration still remains poorly understood.

**Methods:**

Control and injured muscle samples were stained with H&E and immunofluorescent for embryonic myosin heavy chain (eMyHC) at 12, 24, 36, 48, 60, 72, and 84 hours post-injury. Additionally, muscle samples from three mice were immunofluorescent for eMyHC 96 hours post-injury. RNA sequencing and quantitative polymerase chain reaction were performed on 24 mice, including controls and samples at 12-, 24-, and 84-hour post-injury.

**Results:**

Significant upregulation of 516 immune-related and 177 hormone response-related genes was found in this study. Statistical analysis indicated that the number of differentially expressed genes (DEGs) associated with up- and down-regulated immune system-related DEGs was comparable to that of hormone response-related DEGs. The p53 signaling pathway was significantly enriched during early muscle regeneration. Analysis of crucial myogenic genes expression patterns yielded 326 and 320 candidate genes related to satellite cell growth and myofiber formation, respectively. Furthermore, interaction network analysis identified 41 immune factors, including *S100a9*, *Csf3r*, *Cxcl3*, *Ppbp*, *Ccl3*, *Il1rn*, potentially regulating satellite cell activation, migration and proliferation. Likewise, 16 cell adhesion factors (*Col1a2*, *Cdh2*, *Thbs2*, etc.) may be involved in myofiber formation.

**Conclusion:**

This study utilized transcriptomic analysis to identify key candidate genes and biological processes involved in early muscle regeneration. The findings enhance our understanding of the molecular mechanisms underlying muscle repair and offer insights for future therapeutic strategies.

## INTRODUCTION

Skeletal muscle is an orchestrating biological system. Since 1946, the self-repair capacity of skeletal muscle has been characterized [1]. Some studies reveal that muscle regeneration is strictly dependent on the homeostasis between satellite cells and the microenvironment [2]. Specifically, once microenvironment is in a disequilibrium state, muscle repair will be disrupted, leading to muscle disease [3]. Up to now, the effect of microenvironment on muscle regeneration remains unclear.

In adult skeletal muscle, satellite cells maintain quiescent stage and reside in the interstitium between the basement membranes and sarcolemma of myofibers [4]. Once muscle is damaged, satellite cells will be immediately activated and migrated to the lesions. In response to destruction of myofibers, satellite cells need to proliferate and terminally form plurinuclear myotubes to facilitate muscle regeneration. Satellite cell activity is comprehensively regulated by myogenic regulatory factors (MRFs), including *Myf5*, *MyoD*, Myogenin (*MyoG*) and *Mrf4* [5]. In general, the mechanism by which these functional genes regulate muscle development is similar to muscle regeneration [6].

Although the molecular regulation of satellite cell in muscle regeneration is parallel to that during muscle development, microenvironment variation between muscle regeneration and muscle development is strikingly distinct [7]. In particular, immune cells are relatively rare during muscle development while they are largely recruited in muscle regeneration process [8,9], which suggests that immune system (IS) plays an important role in muscle regeneration.

Based on these observations, it is considered that specific regulatory signals present in the early regenerative microenvironment, particularly those involving immune factors, are critical for the activation and proliferation of satellite cells, while distinct transcriptional programs are responsible for controlling myofiber formation at later stages. To better understand the expression patterns of immune factors during the early phase of muscle regeneration and their regulatory effects on myogenic factors, acute muscle injury was induced using cardiotoxin (CTX), and samples were collected at relatively frequent time points. Previous research has found that satellite cells have already migrated and divided at 24 hours post CTX injury (CTX24) [9]. In our study, we chose CTX12 and CTX24 to explore the potential mechanism for satellite cell growth during muscle regeneration. In addition, immunocytochemistry (ICC) assay exhibited that newly formed myofibers extensively formed at CTX84. Therefore, CTX84 was selected to study the molecular mechanism of myofiber formation. We investigated transcriptomes of skeletal muscles at CTX 12, 24 and 84 by Bulk RNA sequencing. These results allowed us to establish underlying molecular networks of microenvironment regulation of early muscle regeneration, which will contribute to a more comprehensive understanding of early muscle regeneration in mammalian skeletal muscle.

## MATERIALS AND METHODS

### Animal and muscle tissue preparation

Eight-week-old male wild-type C57BL/6 mice were purchased from Guangdong Yao Kang Biotechnology for subsequent verification experiments. The total number of animals used for the experiments is 75. In which, 48 were used for hematoxylin and eosin (H&E) staining and immunofluorescence for embryonic MyHC (eMyHC) in controls and injured muscles covering 12 h, 24 h, 36 h, 48 h, 60 h, 72 h and 84 h post injury. H&E staining and immunofluorescence were both in triplicates for each time point. Additionally, 3 mice were just used for immunofluorescence for eMyHC in 96 h injured muscle. 24 mice (controls, 12 h, 24 h, 84 h post injury) were used for RNA-seq and quantitative polymerase chain reaction (qPCR; the total RNA samples of three replicates for each time point were mixed into a pool and each time point is in duplicates). Right side muscles were administered with 100 μL normal saline or 100 μL of 10 μM CTX (Sigma-Aldrich) directly into the middle of anterior tibial muscle (TA). Nitrogen inhalation was used for mice euthanasia. TA muscles were rapidly dissected from injured legs and healthy muscle was used as negative control [2].

### RNA quality assessment, cDNA library construction, and sequencing

Total RNA of TA muscles was extracted by using TRIzol reagent (Life Technologies) according to the manufacturer’s instructions. The RNA integrity and concentration were assessed by NanoDrop ND-1000 (Thermo Fisher Scientific). All the RNA samples were extracted according to the experimental requirements of the Illumina sequencing platform (RNA integrity numbers [RIN]≥7.0 and 28S/18S≥1.0). The quality assessment report of RNA samples was showed in Supplement 1. RNA libraries were constructed and RNA sequencing was performed by Beijing Genomics Institute (BGI) using an Illumina Genome Analyzer. Sequencing reads were generated using Illumina’s Digital Gene Expression Reads Profiling Kit according to the manufacturer’s instructions. These reads were aligned and identified, depended on two reference genomes, Ensemble mouse (000001635.20) and the UniGene database. Clean reads were obtained after removing low quality sequences (there were more than 50% bases with quality lower than 20 in one sequence), reads with more than 5% N bases (bases unknown) and reads containing adaptor sequences, and then they were aligned to the reference database and annotated.

High-throughput sequencing was conducted to produce transcriptome profiles of skeletal muscle at the early stages of muscle regeneration in mice. After filtering adaptor reads, empty reads and low quality reads from the raw data of 8 muscle samples, 5.9±0.06×107 clean reads were remained (Supplement 2). In addition, the base along clean reads had a balanced composition and high quality (Supplements 3A, 3B). Through alignment of clean reads, 82.5±0.81% of clean reads were aligned to reference genome and 74.4±4.7% of clean reads were mapped to UniGene database (Supplement 4). In addition, the reads of 8 muscle samples were evenly distributed on reference genes (Supplement 3C).

### Screening of differentially expressed genes

Reads that could be uniquely mapped to a gene were used to calculate the expression level. Using RSEM tool, fragments Per Kilobase of exon region per Million mapped reads (FPKM) was calculated, whose value can be directly used as the normalized gene expression level. The fold changes (log2 ratio) were estimated according to the normalized gene expression level in each sample. We employed the absolute value of log2 ratio≥1 and probability≥0.8 as the threshold to screen the significantly differentially expressed gene (DEG).

### Gene Ontology analysis and molecular interaction network

To determine the main biological functions, DEGs were annotated by terms of the Gene Ontology (GO) database (http://www.geneontology.org/). GO enrichment analysis of functional significance terms in the GO database was applied using hyper geometric test to find significantly enriched GO terms in DEGs comparing to the genome background. The calculated p-value of GO terms goes through Bonferroni Correction, taking a false discovery rate (FDR)<0.05 as a threshold to screen significantly enriched GO terms in DEGs. In addition, GO functional analysis of candidate gene was carried out by DAVID analysis (http://david.abcc.ncifcrf.gov/). The molecular interaction network of candidate genes was constructed through STRING (http://string-db.org/) and Cytoscape 3.1.0 (http://www.cytoscape.org/). High confidence (0.7) was used in STRNG analysis.

### Pathway enrichment analysis

Genes usually interact with each other to take part in certain biological functions. Pathway functional analysis of up-regulated DEGs was carried out by DAVID analysis (http://david.abcc.ncifcrf.gov/). Up-regulated DEGs of our comparison groups were classified into many significant enrichment pathways. Significantly enriched pathway was defined by p value<0.05.

### Real-time quantitative polymerase chain reaction

11 genes were chosen to validate the accuracy of RNA sequencing. The remaining RNA from sequencing was used. cDNA was generated from 1 μg of total RNA by reverse transcription system (Promega) according to the manufacturer’s instructions. The qPCR reaction was carried out in the LightCycler 480 II system (Roche), using 2×SYBR Green Realtime PCR Master Mix (Takara) according to the manufacturer’s instructions. The analytic method of 2^−ΔΔCt^ was used. The Spearman’s correlation coefficient was calculated for the gene to assess the consistency between qPCR and RNA sequencing. Supplement 5 lists the gene-specific primers for gene expression.

### Histology and immunocytochemistry of injured muscle

TA was dissected from injured muscle, fixed in 4% paraformaldehyde (PFA) for 24 h at 4°C, then embedded with paraffin. Paraffin-embedded muscle was sectioned at 4 μm. After deparaffinating, muscle section was stained by H&E. Cell nucleus was stained by hematoxylin in 1 min, washed by 95% ethyl alcohol. Cytoplasm was stain by eosin in 20 seconds, washed by 95% ethyl alcohol, finally washed in PBS and mounted by neutral balsam. H&E staining images of muscle sections were taken by a fluorescence inverse microscope (ZEISS).

For ICC, OCT-embedded TA muscles were sectioned at 6 μm, fixed for 10 minutes in acetone at −20°C, permeabilized with 0.5% of Triton X-100 in PBS for 15 minutes followed by being blocked with Hydrogen Peroxide Block for 15 minutes, washed by PBS, blocked with Rodent Block (ab127055; Abcam) for 30 minutes, then incubated with primary antibodies against eMyHC (F1.652-b; DSHB) and LaminA2 (ab11576; Abcam) for 1 hour at room temperature. After removing primary antibodies and washing, muscle sections were incubated by anti-Mouse IgG secondary antibodies (#4409, CST) for eMyHC and anti-Rat IgG secondary antibodies (#4418S, CST) for LaminA2 in 10 min at room temperature. After being washed by PBS, sections were stained by DAPI for 2 minutes. Lastly the muscle sections were mounted by anti-fade Mounting Medium. Images were taken by confocal microscopy (Leica).

### Statistical analysis

For screening the DEGs of each comparison group, we employed the absolute value of log2 ratio≥1 and probability≥0.8 as the threshold. FDR<0.05 was applied to screen significantly enriched GO terms in DEGs. In addition, p value<0.05 was used to define significantly enriched pathways. The Spearman’s correlation coefficient was calculated for the gene to assess the consistency between qPCR and RNA sequencing. Significant correlation was accepted when p<0.01.

## RESULTS

### Great changes in morphology and genes expression profiles of injured muscles

**Figure 1:**
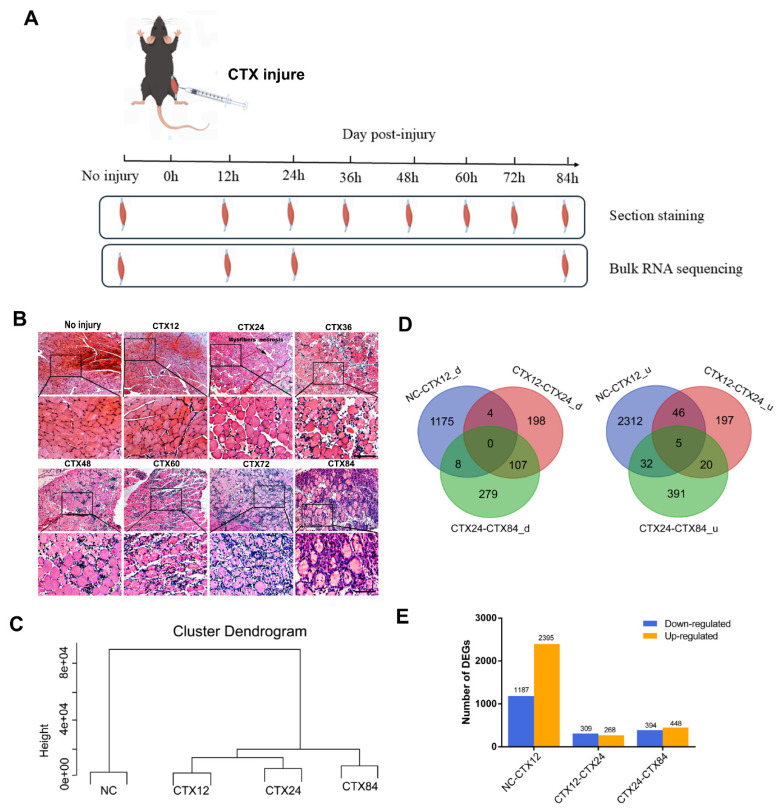
Global transcriptome analysis of the injured muscles during early muscle regeneration. (A) Mice were injected with 100 μL normal saline or 100 μL of 10 μM cardiotoxin (CTX) into tibialis anterior (TA) muscles. TA muscles were isolated at different time points as indicated. (B) Morphological features of injured muscle. All experiments were repeated three times with similar results. Representative images are shown. Scale bar = 100 μm. (C) Hierarchical classification analysis shows reproducibility of transcriptome profiles of muscles at each time point (sampled in duplicates) and divides the four time points into three major branches. (D) Venn Chart analysis of significantly up- and down-regulated gene numbers at adjacent stages. d means down-regulated genes, u means up-regulated genes. (E) Histogram analysis of up- and down-regulated gene numbers.

To characterize the morphology of CTX-injured muscles, we performed H&E staining to characterize the morphologies of negative controls (NC) and injured muscles covering 12 h, 24 h, 36 h, 48 h, 60 h, 72 h and 84 h post injury (Figure 1A). At CTX12, the number of nucleus was obviously more than that in NC sample, which suggested that inflammatory cells had already migrated and proliferated. Subsequently, myofibers underwent necrosis and edematous (Figure 1B). Notably, the H&E results at CTX36, CTX48, and CTX60 closely resembled those observed at CTX24, with persistent necrosis and inflammatory infiltration. 8 muscle samples in total were grouped into four categories, of which NC sample was far away from injured muscle samples, the CTX12 sample was closely next to CTX24 sample and far away from CTX84 sample (Figure 1C).

To uncover the genes involved in early muscle regeneration, we totally obtained 4,774 DEGs with the absolute value of log2 fold change≥1 and probability≥0.8 as the threshold. In Figure 1D, totally 1,771 DEGs were up-regulated and 3,003 DEGs were down-regulated in three comparison groups. Additionally, in NC-CTX12 comparison group, 2,395 up-regulated and 1187 down-regulated genes were identified. In CTX12-CTX24 comparison group, we found 268 up-regulated and 309 down-regulated genes while 448 up-regulated and 394 down-regulated genes in CTX24-CTX84 comparison group (Figure 1E). These results indicated that the number of DEGs in NC-CTX12 is far more than that in CTX12-CTX24 or CTX24-CTX84, suggesting that the injured-activated genes at CTX12 are the most robust during early muscle regeneration.

### Specific pathways involved in different stages of early muscle regeneration

**Figure 2:**
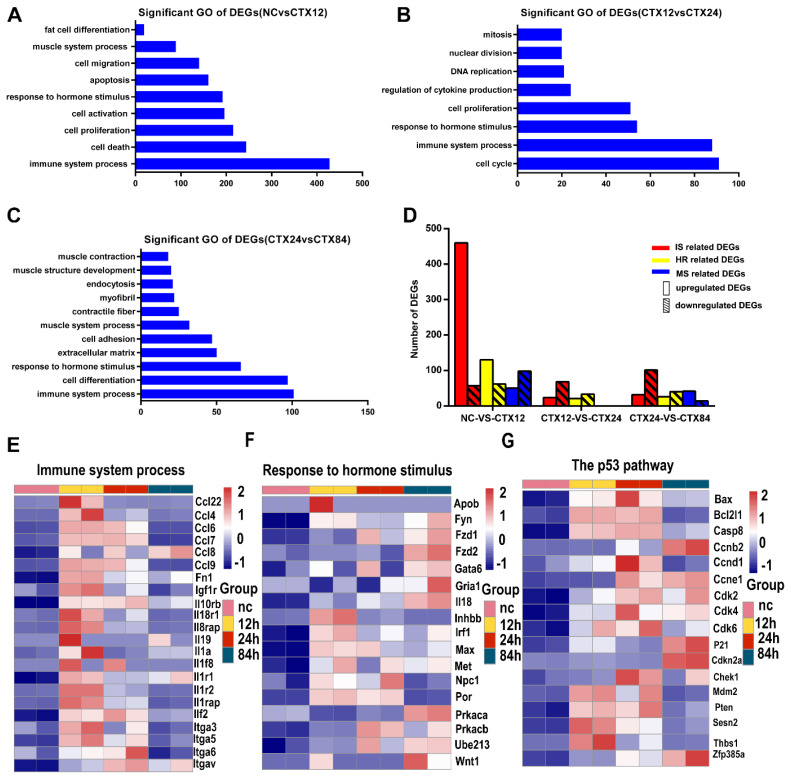
Number of DEGs of immune system (IS), hormone response (HR) and muscle system (MS) in each comparison group. (A–C) Comparison of significant GO biological processes of significantly enriched GO terms of DEGs for each comparison group during early muscle regeneration (FDR<0.05). (D) Statistical analysis of up- and down-regulated DEGs of GO terms that include IS, HR and MS. Note that the up- and down-regulated DEG of MS in CTX12-CTX24 group is not found. (E) Heatmap of genes involved in the IS process. (F) Heatmap of genes involved in the response to hormones stimulus. (G) Heatmap of genes involved in the p53 signaling pathway. GO, Gene Ontology; DEG, differentially expressed gene; FDR, false discovery rate.

To identify signaling pathways involved in early muscle regeneration, DEGs were mapped to the KEGG database. Significantly enriched functional pathways of up-regulated DEGs in three comparison groups were presented in Table 1. In NC-CTX12 comparison group, MAPK, Notch and chemokine signaling pathways were dramatically activated, which have previously been identified to regulate satellite cell proliferation [10,11]. DNA replication and cell cycle related genes were enriched in CTX12-CTX24 comparison group. Focal adhesion and extracellular matrix (ECM)-receptor interaction pathways were enriched in CTX24-CTX84 comparison group. More importantly, p53 signaling pathway was always significantly enriched in three comparison groups, which indicated that p53 pathway was involved in entire early process of muscle regeneration process and plays important role (Figure 2G).

### Both immune system and hormone response are activated in early muscle regeneration

To further study the biological function of DEGs during early muscle regeneration, the DEGs were classified by GO analysis. Bar graph shows that DEGs were mostly enriched in IS, cell proliferation and cell activation in NC-CTX12 group (Figure 2A). In CTX12-CTX24 group, DEGs were enriched in cell cycle, cell proliferation and IS processes (Figure 2B). In CTX24-CTX84 group, DEGs were enriched in IS, cell differentiation, muscle system (MS) and cell adhesion (Figure 2C). Interestingly, DEGs involved in IS and hormone response (HR) were shared in three comparison groups. Further, we totally found 516 immune related and 177 HR related up-regulated DEGs during early muscle regeneration. These implied that IS and HR were activated for regulating early muscle regeneration. Furthermore, up- and down-regulated DEGs of MS, IS and HR were classified. Statistical analysis showed that the number of up-regulated IS and HR related DEGs was more than that of down-regulated DEGs while the number of up-regulated MS related DEGs was less than that of down-regulated DEGs in NC-CTX12 group. However, the number of up-regulated IS and HR related DEGs was less than that of down-regulated DEGs in CTX12-CTX24 and CTX24-CTX84 groups while the number of up-regulated MS related DEGs was more than that of down-regulated DEGs in CTX24-CTX84 group (Figure 2D). These results indicated that the difference of DEG number between up- and down-regulated IS was similar with that of HR, but contrary to MS (Figures 2E, 2F). As IS is known to trigger muscle regeneration, we assumed that hormone regulation may also play an important role in early muscle regeneration.

### Candidate immune-related genes are associated with satellite cell growth

**Figure 3:**
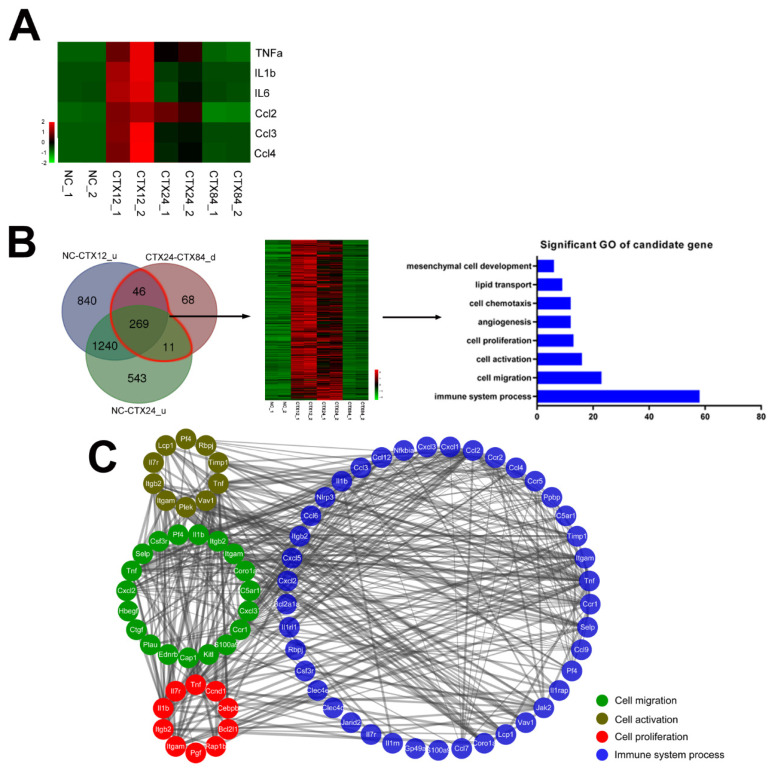
Enrichment of candidate immune factors that potentially regulated satellite cell growth. (A) Heatmap diagrams of crucial immune factors are represented using a green-to-red color scale. (B) Genes that had similar expression patterns were screened and GO analysis of these genes was taken (FDR<0.05). u means significantly up-regulated genes. d means significantly down-regulated genes. Genes that had similar expression patterns are circled in red line, expression patterns of which are shown by Heatmap diagrams. (C) Molecular interaction network analysis of relationship among immune system, cell migration, activation and proliferation. The interaction network of these genes was predicted using STRING website and illustrated by Cytoscape software. Four cycles are shown in this figure as cell proliferation, activation and migration genes are listed at left; immune system genes are listed at right. Thicker line represents stronger interaction. GO, Gene Ontology; FDR, false discovery rate.

To comprehensively explore how IS regulates satellite cell growth, 6 key immune factors regulating satellite cell proliferation were selected and these gene expression patterns were presented in heat maps. The heat maps illustrated that they displayed highly similar expression patterns (Figure 3A). Genes with similar expression patterns usually have similar biological function. Therefore, according to aforementioned expression patterns, 326 candidate genes were obtained. Then enrichment analysis was performed to inspect whether these genes shared common features. In Figure 3B, these candidate genes were mostly associated with IS, cell proliferation, activation and migration. The interaction network of these genes was predicted using STRING website and illustrated by Cytoscape software. It showed that 41 immune factors were identified and closely interconnected with genes involved in cell proliferation, activation and migration (Figure 3C), which implied that these immune factors potentially regulated satellite cell activation, migration and proliferation. Further, we calculated the fold change of 41 candidate immune factors expression level in injured muscle compared to NC (Supplement 6) and found that fold changes of S100a9, Csf3r, Cxcl3, Ppbp, Ccl3 and Il1rn were over 30 times, indicating that these immune factors may play more important role in satellite cell regulation during muscle regeneration.

### Candidate cell adhesion genes are associated with myofiber formation

**Figure 4:**
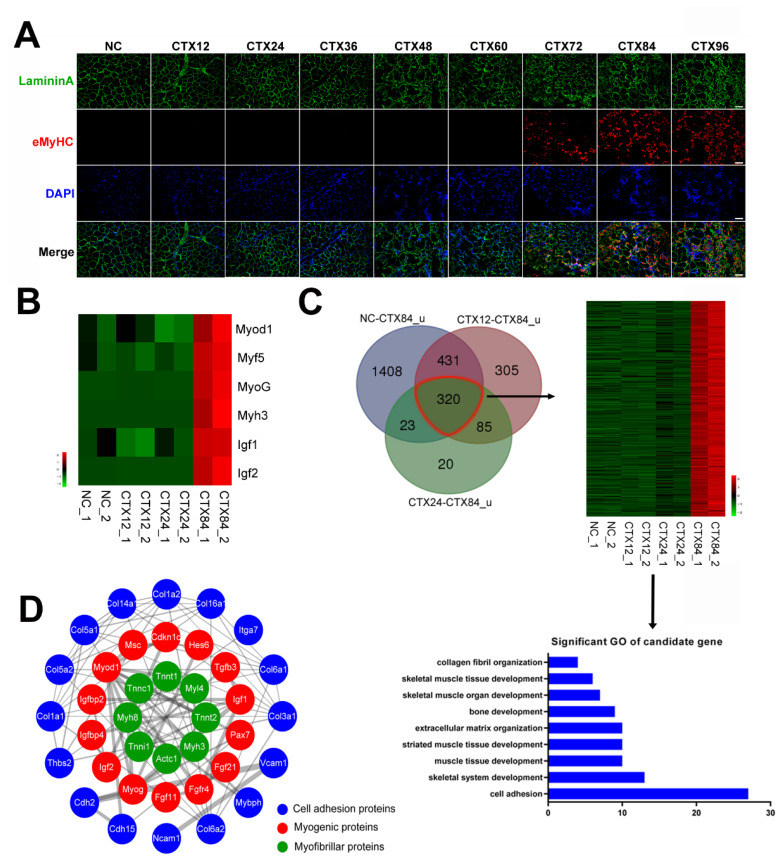
Enrichment of candidate cell adhesion factors involved in regulation of myofiber formation. (A) Immunofluorescence for eMyHC^+^ myofibers during early muscle regeneration. eMyHC^+^ myofibers were stained in red, nuclei were stained in blue by DAPI, LamininA were stained in Green. Scale bar = 100 μm. (B) Heatmap diagrams of crucial myogenic factors expression level are represented using a green-to-red color scale. (C) Genes that have similar expression patterns were screened and GO analysis of these genes was taken (FDR<0.05). u means significantly up-regulated genes. Genes that had similar expression patterns are circled in red line, expression patterns of which are shown by Heatmap diagrams. (D) Molecular interaction network analysis between adhesion and myogenesis. The interaction network of these genes was predicted using STRING website and illustrated by Cytoscape software. Three cycles are shown in this figure as follows: myofibrillar proteins are in green cycle, myogenic regulating factors are in red cycle, cell adhesion proteins are in blue cycle. Thicker line represents stronger interaction. eMyHC, embryonic MyHC; GO, Gene Ontology; FDR, false discovery rate.

To characterize the formation of newly formed myofibers during early muscle regeneration, eMyHC, a marker of newly formed myofibers, was detected by ICC. The results showed that eMyHC was extensively expressed in skeletal muscle at CTX84 (Figure 4A), suggesting that genes involved in myofiber formation were activated at CTX84. To further investigate candidate genes governing myofiber formation, key myogenic factors were chosen and their mRNA expression patterns during muscle early regeneration were presented by heat maps. It showed that these genes were significantly up-regulated at CTX84 (Figure 4B). Further, 320 candidate genes that shared similar expression patterns with myogenic factors were obtained. GO analysis indicated that most of these genes were associated with cell adhesion and muscle development (Figure 4C). STRING prediction result showed that these interacting proteins were classified into three kinds of functional proteins (myofibrillar proteins, myogenic proteins and cell adhesion proteins) (Figure 4D). Obviously, these cell adhesion proteins influence the formation of muscle fibers by regulating myogenic proteins during muscle regeneration process.

### Validation of differentially expressed genes by quantitative polymerase chain reaction

**Figure 5:**
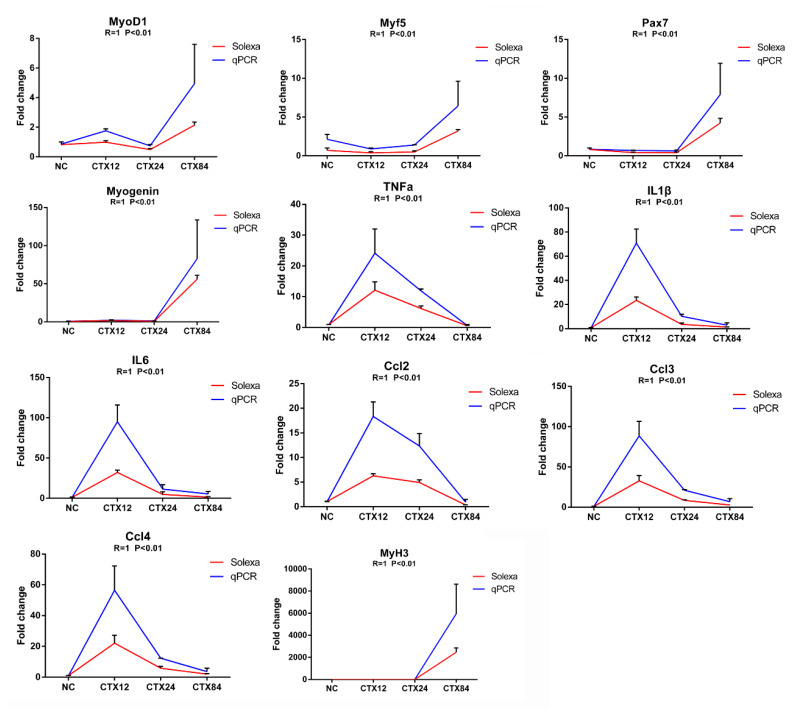
Validation of RNA sequencing data with qPCR. The vertical axis indicates the fold changes of expression level by RNA or qPCR compared to the NC. The horizontal axis indicates the samples for the control and injured muscles. R-value represents the Spearman’s correlation between the two methods. Error bars represent the mean±SEM, n = 2 for RNA sequencing and n = 3 for qPCR. qPCR, quantitative polymerase chain reaction; SEM, standard error of the mean.

To validate RNA sequencing data, relative mRNA expression of 11 DEGs were detected by qPCR (Figure 5). Specifically, *Myod1*, *Myf5*, *Pax7* and *MyoG* are crucial regulators in myogenesis and muscle regeneration [6]. TNFa, IL1β, IL6, Ccl2, Ccl3 and Ccl4 play important roles in regulation of satellite cell proliferation [7]. In addition, eMyHC involved in regeneration of myofibers upon injury is encoded by MyH3 [12]. The correlation between qPCR results and RNA sequencing data of DEGs was carried out by Spearman’s correlation coefficient analysis and the result showed that the correlation coefficient (R) was greater than 0.8 in all the genes, indicating that expression profiles of these genes have high consistency between two methods.

## DISCUSSION

During muscle regeneration, microenvironment has been reported to influence the satellite cell activity, terminally inducing muscle regeneration [13,14]. However, the mechanism by which microenvironment regulate satellite cell activity and myofiber formation still remains poorly understood.

### p53 pathway involved in the whole early muscle regeneration

In our study, GO analysis of significantly up-regulated DEGs during early muscle regeneration indicated that several pathways known to be closely involved in regulation of satellite cell activity were enriched (Table 1). Specifically, chemokine and notch signaling have been reported to promote satellite cell activation, migration and proliferation [15–17]. Previous reports have shown that p53 acts as suppressor to regulate cell proliferation and apoptosis [18]. Moreover, p53 signaling pathway is activated during myogenesis and p53 interference prevents C2C12 myoblast differentiation [19]. Furthermore, recent findings have shown that mechano-signaling via Piezo1 prevents the activation of p53 and its associated senescence program in muscle stem cells, thereby promoting regenerative capacity following injury [20]. In our data, p53 pathway was enriched throughout the stages of early muscle regeneration, as evidenced by the sustained upregulation of key target genes (Figure 2G), indicating that it is very important during the entire early muscle regeneration.

### Hormones may play a trigger role in early muscle regeneration

Hormones, a class of secreted factors including adrenal cortical hormone, sex hormone, thymin, oxytocin and so on, plays important roles in development and physiological metabolism [21]. Recently, several studies have focused on the effects of hormones on muscle regeneration. Systemic administration of oxytocin promotes satellite cell proliferation for muscle regeneration, while satellite cell proliferation is impaired by inhibiting oxytocin signaling [22]. In addition, inactivation of thyroid hormone (T3) by type 3-deiodinase (D3) improves muscle regeneration through surviving satellite cell proliferation [23]. Decreased androgen impaired muscle regeneration [24]. Moreover, Liao et al [25] reported that estrogen promotes the polarization of macrophages toward an anti-inflammatory M2 phenotype and modulates T cell responses in injured muscle, thereby facilitating repair. Similarly, insulin and amino acids cooperatively activate the mTORC1 pathway in skeletal muscle, supporting regeneration through converging hormonal and nutritional signals [26]. Based on our transcriptome analysis, the number of DEGs between up- and down-regulated IS was similar with that of HR (Figure 2D). It was well known that IS is a key process in early muscle regeneration. Therefore, it allowed us to think that hormones may play an analogical role in early muscle regeneration, which could be a novel strategy for the treatment of muscle disease.

### Candidate immune genes arouse satellite cells for early regeneration

After muscle injury, the key inflammatory cells are rapidly activated and secrete immune factors to govern the microenvironment where satellite cell locates [27]. It has been determined that several important immune factors are able to regulate satellite cell activity for muscle regeneration. In particular, chemokines, including Ccl2, Ccl3 and Ccl4, directly promote myoblast proliferation by regulating the phosphorylation of ERK1/2 [16]. In parallel, the granulocyte colony-stimulating factor receptor (Csf3r) facilitates muscle repair by mediating neutrophil recruitment [28], while the interleukin-1 receptor antagonist (Il1rn) fine-tunes the inflammatory response to prevent excessive tissue damage [29]. IL1β and TNFα directly activate NF-κb to enhance the proliferation of satellite cells through promoting expression of cyclinD [30]. In addition, knocking out IL6 impairs satellite cell proliferation by decreasing STAT3 phosphorylation and cyclinD1 expression [31]. During early muscle regeneration, these immune factors had similar expression patterns (Figure 3A), which suggested that immune factors that have similar patterns may regulate satellite cell growth during early muscle regeneration.

To explore more candidate immune genes regulating satellite cell growth, we screened 58 immune genes that shared similar expression patterns with immune factors mentioned above (Figure 3B). Interaction network presented those 41 immune factors showed strong relationship with cell migration, activation and proliferation factors (Figure 3C). Further, we focused on *S100a9*, *Csf3r*, *Cxcl3*, *Ppbp*, *Ccl3*, *Il1rn* that may be crucial to migration, activation and proliferation of satellite cells because of their high expression level during early muscle regeneration. S100a9 and Ccl3 have been identified to regulate C2C12 cell proliferation [16,32]. In addition, Csf3r mediates hematopoietic progenitor mobilization in mice [33]. Cxcl3 regulates smooth muscle cell migration [34], and Ppbp facilitates intravascular leukocyte migration directly [35]. Therefore, *Csf3r*, *Cxcl3*, *Ppbp* may be also involved in regulation of satellite cell migration. Interleukin 1 receptor antagonist (Il1rn) inhibits the activation of NF-κb which is a crucial factor in satellite cell proliferation, suggesting that Il1rn could regulate satellite cell proliferation through NF-κb. Together with our study, these candidate immune genes may be involved in satellite cell activity during early muscle regeneration.

### Cell adhesion factors are associated with myofiber formation

**Figure 6:**
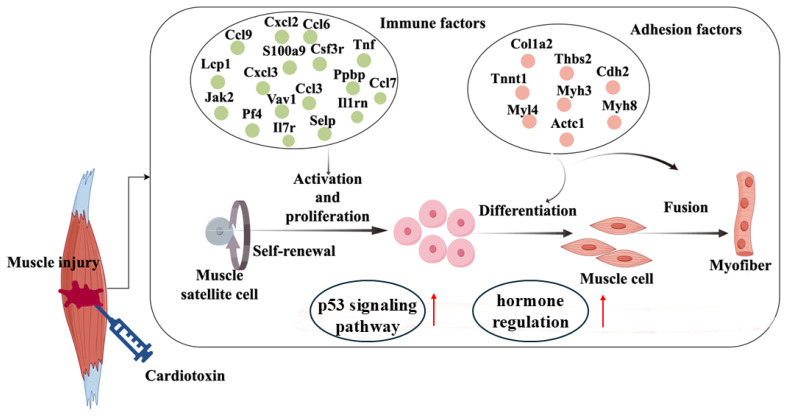
Key candidate genes and biological processes involved in early muscle regeneration.

In our study, newly formed myofibers were widely formed at CTX84, meanwhile, the mRNA level of muscular regulators was significantly up-regulated (Figures 4A, 4B). Based on the expression patterns of muscular regulators in Figure 4B, we screened 320 genes, of which 27 genes were cell adhesion factors (Figure 4C), meanwhile ECM-receptor interaction and focal adhesion signaling were activated at CTX84 (Table 1), implying that 27 cell adhesion factors were potentially involved in myofiber formation. Moreover, molecular network showed that 16 cell adhesion proteins were related to myogenic regulators and myofibrillar proteins (Figure 4D). Previous report has found that cadherin regulates phosphorylation of β-catenin to influence myoblast differentiation [36], which implied that cdh2 and cdh15 may regulate myoblast differentiation during muscle regeneration. In addition, ablation of fibroblast in skeletal muscle promotes satellite cell premature differentiation, leading to impair muscle regeneration [37]. Collagen is mostly secreted by fibroblasts and acts as framework for guiding satellite cells to facilitate muscle regeneration [9,38,39]. Further, knocking out Col6a1 in mice attenuates satellite cell proliferation, resulting in muscle regeneration deficiency [5]. These findings suggested that collagen plays crucial roles in muscle regeneration. In our molecular network, 9 collagen proteins were found, which may play important roles in early muscle regeneration. In addition, Thbs2, a member of ECM proteins, can mediate collagen fibrillogenesis, which hinted that thbs2 could indirectly influence muscle regeneration through regulating collagen formation. Our results indicated that candidate cell adhesion factors may be involved in muscle regeneration (Figure 6).

## CONCLUSION

In conclusion, our transcriptome analysis revealed that early muscle regeneration is orchestrated by multiple interconnected pathways, including immune response, hormone signaling, p53 pathway activation, and cell adhesion processes. We identified key candidate genes such as *Ccl3*, *S100a9*, *Csf3r*, and *Il1rn*, which are potentially involved in satellite cell migration, activation, and proliferation. In addition, several cell adhesion molecules and ECM components, including *cdh2*, *cdh15*, *Col6a1*, and *Thbs2*, were found to be associated with myofiber formation. These findings provide novel insights into the molecular regulation of early muscle regeneration and highlight potential targets for therapeutic strategies aimed at enhancing muscle repair.

## Figures and Tables

**Table 1:** Significant functional pathways of up-regulated DGEs of three comparison groups

Group compared	Pathway name	DEGs	p-value
NC vs. CTX12	Chemokine signaling pathway	83	3.80E-23
	Toll-like receptor signaling pathway	45	1.30E-12
	Apoptosis	39	8.90E-11
	B cell receptor signaling pathway	33	3.80E-08
	Jak-STAT signaling pathway	49	1.30E-07
	T cell receptor signaling pathway	41	1.80E-07
	MAPK signaling pathway	63	1.40E-04
	p53 signaling pathway	20	4.90E-03
	Notch signaling pathway	15	1.30E-02
CTX12 vs. CTX24	DNA replication	17	3.20E-20
	Cell cycle	20	1.40E-13
	Mismatch repair	6	2.50E-05
	Nucleotide excision repair	6	6.90E-04
	p53 signaling pathway	7	9.60E-04
CTX24 vs. CTX84	ECM-receptor interaction	13	1.90E-07
	Dilated cardiomyopathy	12	4.40E-06
	Focal adhesion	14	3.70E-04
	p53 signaling pathway	7	3.90E-03
	Cardiac muscle contraction	7	7.00E-03

DEG, differentially expressed gene.

## SUPPLEMENTARY MATERIAL

**Supplement 1**. The quality assessment of RNA samples.

**Supplement 2**. Raw data statistic of each stage in the control and injured muscles.

**Supplement 3**. Assessment of Solexa sequencing quality.

**Supplement 4**. Alignment statistics of Solexa sequencing of muscle samples.

**Supplement 5**. Primer information of the genes in real-time fluorescent qPCR.

**Supplement 6**. Fold change of 41 candidate immune factors expression level between healthy muscle and injured muscle.
