# Supplementary material for: Transcriptome analysis of injured muscle identifies new candidate genes for satellite cell growth and myofiber formation during early muscle regeneration
Source: Anim Biosci. 2025 Aug 12;39(2):240859. doi: 10.5713/ab.24.0859 (PMC12877386; doi:10.5713/ab.24.0859)

Supplementary 1. The quality assessment of RNA samples.

Sample name	Concentration (ng/µL)	Volume (µL)	Total （µg）	RIN	28S/18S
NC_1	186	20	3.72	7.8	1.1
NC_2	368	50	18.4	8.8	1.5
CTX12_1	198	20	3.96	8.1	1.3
CTX12_2	67	20	1.34	8.4	1.3
CTX24_1	444	20	8.88	7.8	1.3
CTX24_2	238	40	9.52	9.2	1.6
CTX84_1	206	20	4.12	8.3	1.4
CTX84_2	96	20	1.92	7.5	1.6

Raw Data Statistics

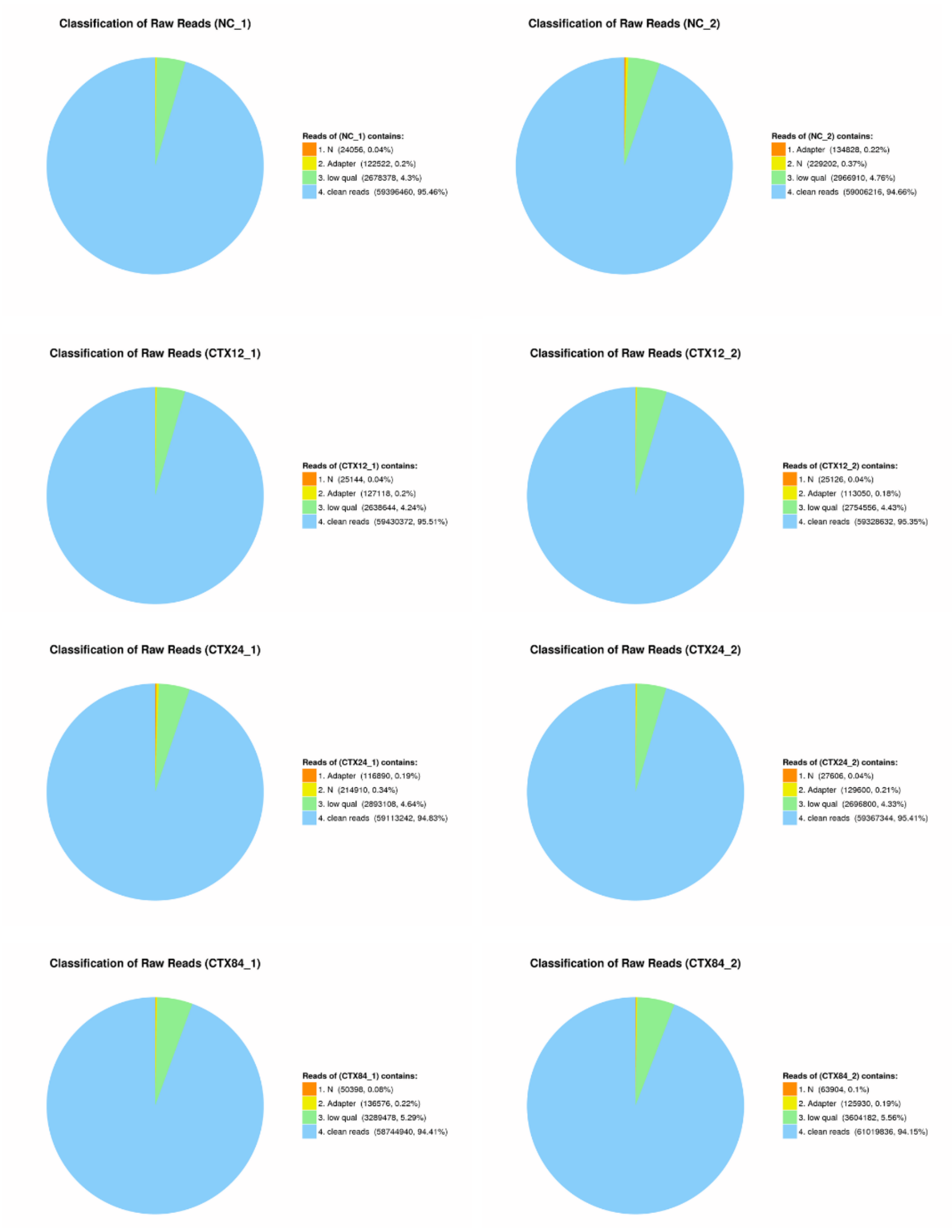

Supplement: Supplementary file 1 [file ab-24-0859-Supplementary-1.pdf]
