# Supplementary material for: Transcriptome analysis of injured muscle identifies new candidate genes for satellite cell growth and myofiber formation during early muscle regeneration
Source: Anim Biosci. 2025 Aug 12;39(2):240859. doi: 10.5713/ab.24.0859 (PMC12877386; doi:10.5713/ab.24.0859)
Supplement: Supplementary file 2 [file ab-24-0859-Supplementary-2.pdf]

Supplementary 2. Raw data statistic of each stage in the control and injured muscles. "N" indicates reads in which unknown bases are more than 10%; "Adapter" indicates reads with adapters; "Low qual" indicates low quality reads (the percentage of low quality bases is over 50% in a read, we define the low quality base to be the base whose sequencing quality is no more than 10); "Clean reads" indicates the remaining reads, after filtering above three kinds of "dirty" raw reads, and were used for downstream bioinformatics analysis.

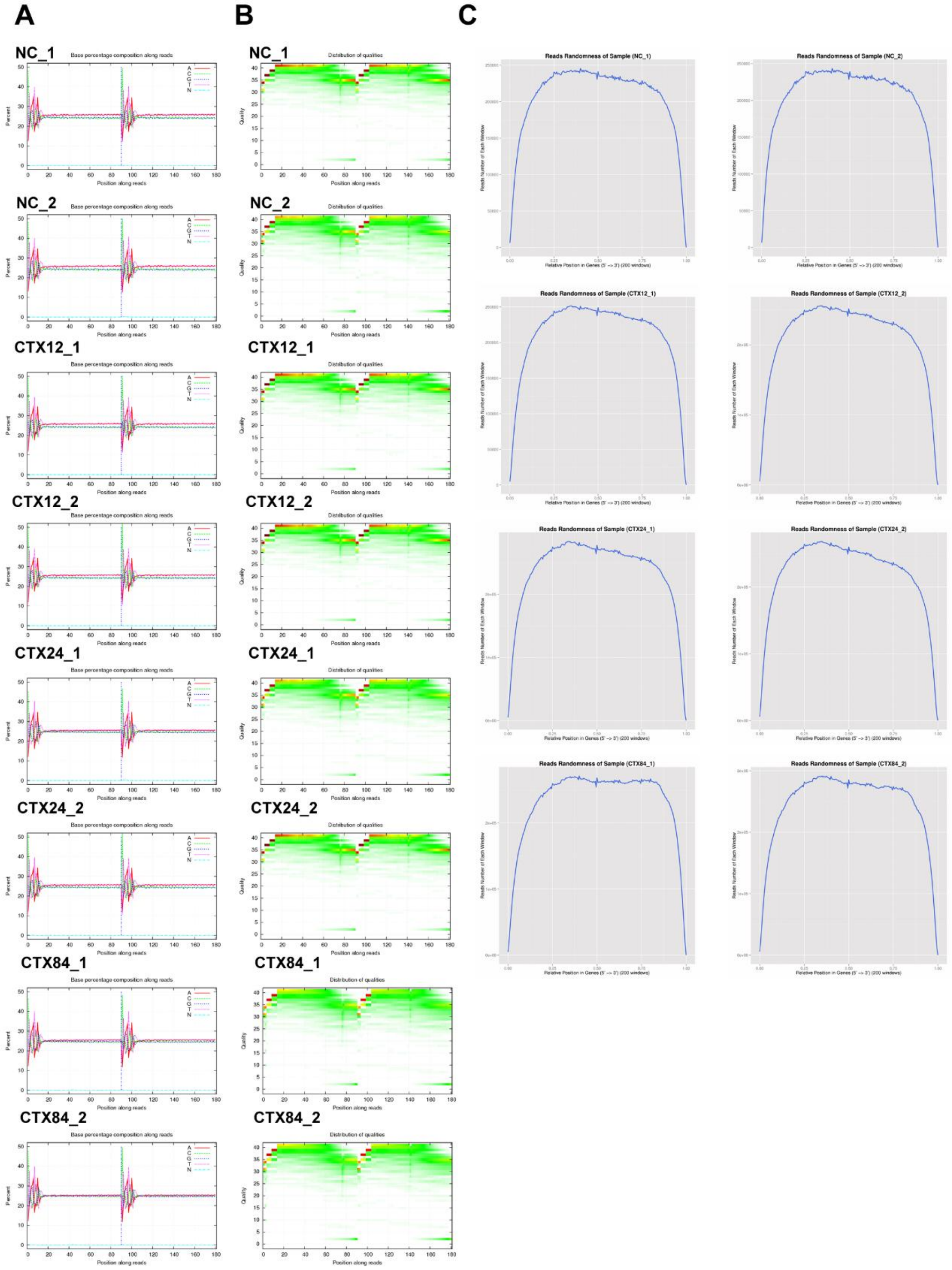

Supplementary 3. Assessment of Solexa sequencing quality. (A) Base composition of clean
