# Supplementary material for: Transcriptome analysis of injured muscle identifies new candidate genes for satellite cell growth and myofiber formation during early muscle regeneration
Source: Anim Biosci. 2025 Aug 12;39(2):240859. doi: 10.5713/ab.24.0859 (PMC12877386; doi:10.5713/ab.24.0859)
Supplement: Supplementary file 3 [file ab-24-0859-Supplementary-3.pdf]

Supplementary 2. Raw data statistic of each stage in the control and injured muscles. “N” indicates reads in which unknown bases are more than 10%; “Adapter” indicates reads with adapters; “Low qual” indicates low quality reads (the percentage of low quality bases is over 50% in a read, we define the low quality base to be the base whose sequencing quality is no more than 10); “Clean reads” indicates the remaining reads, after filtering above three kinds of “dirty” raw reads, and were used for downstream bioinformatics analysis.

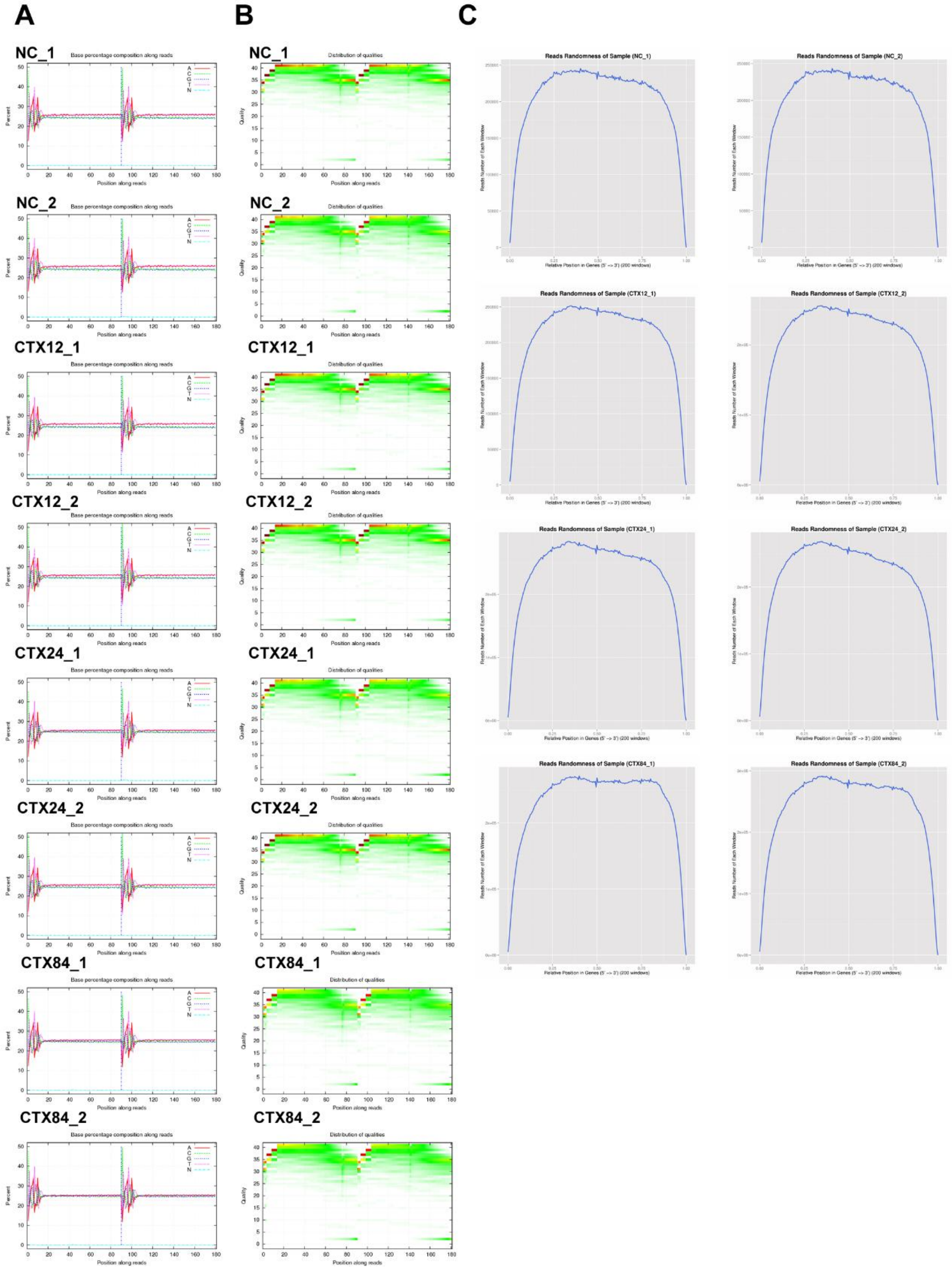

Supplementary 3. Assessment of Solexa sequencing quality. (A) Base composition of clean

reads. (B) Quality distribution of bases along reads. (C) Distributions of reads on reference genes.

Supplementary 4. Alignment statistics of Solexa sequencing of muscle samples

Map to Genome	Reads Number	Percent	Map to Gene	Reads Number	Percent
**Mapping statistics of sample NC_1**					
Total Reads	59396460	100.00%	Total Reads	59396460	100.00%
Total BasePairs	5345681400	100.00%	Total BasePairs	5345681400	100.00%
Total Mapped Reads	48507595	81.67%	Total Mapped Reads	41427788	69.75%
Perfect Match	39936821	67.24%	Perfect Match	35525216	59.81%
Mismatch	8570774	14.43%	Mismatch	5902572	9.94%
Unique Match	40613599	68.38%	Unique Match	38704408	65.16%
Multi-position Match	7893996	13.29%	Multi-position Match	2723380	4.59%
Total Unmapped Reads	10888865	18.33%	Total Unmapped Reads	17968670	30.25%
**Mapping statistics of sample NC_2**					
Total Reads	59006216	100.00%	Total Reads	59006216	100.00%
Total BasePairs	5310559440	100.00%	Total BasePairs	5310559440	100.00%
Total Mapped Reads	48336632	81.92%	Total Mapped Reads	41214774	69.85%
Perfect Match	38600496	65.42%	Perfect Match	34157030	57.89%
Mismatch	9736136	16.50%	Mismatch	7057744	11.96%
Unique Match	39820050	67.48%	Unique Match	38528204	65.30%
Multi-position Match	8516582	14.43%	Multi-position Match	2686570	4.55%
Total Unmapped Reads	10669584	18.08%	Total Unmapped Reads	17791440	30.15%
**Mapping statistics of sample CTX12_1**					
Total Reads	59430372	100.00%	Total Reads	59430372	100.00%
Total BasePairs	5348733480	100.00%	Total BasePairs	5348733480	100.00%
Total Mapped Reads	49834741	83.85%	Total Mapped Reads	42363292	71.28%
Perfect Match	40704717	68.49%	Perfect Match	36011018	60.59%
Mismatch	9130024	15.36%	Mismatch	6352274	10.69%
Unique Match	41940795	70.57%	Unique Match	39818194	67.00%
Multi-position Match	7893946	13.28%	Multi-position Match	2545098	4.28%
Total Unmapped Reads	9595631	16.15%	Total Unmapped Reads	17067078	28.72%
**Mapping statistics of sample CTX12_2**					
Total Reads	59328632	100.00%	Total Reads	59328632	100.00%
