# Supplementary material for: Transcriptome analysis of injured muscle identifies new candidate genes for satellite cell growth and myofiber formation during early muscle regeneration
Source: Anim Biosci. 2025 Aug 12;39(2):240859. doi: 10.5713/ab.24.0859 (PMC12877386; doi:10.5713/ab.24.0859)
Supplement: Supplementary file 4 [file ab-24-0859-Supplementary-4.pdf]

reads. (B) Quality distribution of bases along reads. (C) Distributions of reads on reference genes.

Supplementary 4. Alignment statistics of Solexa sequencing of muscle samples

Map to Genome	Reads Number	Percent	Map to Gene	Reads Number	Percent
**Mapping statistics of sample NC_1**					
Total Reads	59396460	100.00%	Total Reads	59396460	100.00%
Total BasePairs	5345681400	100.00%	Total BasePairs	5345681400	100.00%
Total Mapped Reads	48507595	81.67%	Total Mapped Reads	41427788	69.75%
Perfect Match	39936821	67.24%	Perfect Match	35525216	59.81%
Mismatch	8570774	14.43%	Mismatch	5902572	9.94%
Unique Match	40613599	68.38%	Unique Match	38704408	65.16%
Multi-position Match	7893996	13.29%	Multi-position Match	2723380	4.59%
Total Unmapped Reads	10888865	18.33%	Total Unmapped Reads	17968670	30.25%
**Mapping statistics of sample NC_2**					
Total Reads	59006216	100.00%	Total Reads	59006216	100.00%
Total BasePairs	5310559440	100.00%	Total BasePairs	5310559440	100.00%
Total Mapped Reads	48336632	81.92%	Total Mapped Reads	41214774	69.85%
Perfect Match	38600496	65.42%	Perfect Match	34157030	57.89%
Mismatch	9736136	16.50%	Mismatch	7057744	11.96%
Unique Match	39820050	67.48%	Unique Match	38528204	65.30%
Multi-position Match	8516582	14.43%	Multi-position Match	2686570	4.55%
Total Unmapped Reads	10669584	18.08%	Total Unmapped Reads	17791440	30.15%
**Mapping statistics of sample CTX12_1**					
Total Reads	59430372	100.00%	Total Reads	59430372	100.00%
Total BasePairs	5348733480	100.00%	Total BasePairs	5348733480	100.00%
Total Mapped Reads	49834741	83.85%	Total Mapped Reads	42363292	71.28%
Perfect Match	40704717	68.49%	Perfect Match	36011018	60.59%
Mismatch	9130024	15.36%	Mismatch	6352274	10.69%
Unique Match	41940795	70.57%	Unique Match	39818194	67.00%
Multi-position Match	7893946	13.28%	Multi-position Match	2545098	4.28%
Total Unmapped Reads	9595631	16.15%	Total Unmapped Reads	17067078	28.72%
**Mapping statistics of sample CTX12_2**					
Total Reads	59328632	100.00%	Total Reads	59328632	100.00%

Total BasePairs	5339576880	100.00%	Total BasePairs	5339576880	100.00%
Total Mapped Reads	49598237	83.60%	Total Mapped Reads	42702868	71.98%
Perfect Match	40613571	68.46%	Perfect Match	36454959	61.45%
Mismatch	8984666	15.14%	Mismatch	6247909	10.53%
Unique Match	42065934	70.90%	Unique Match	40159620	67.69%
Multi-position Match	7532303	12.70%	Multi-position Match	2543248	4.29%
Total Unmapped Reads	9730395	16.40%	Total Unmapped Reads	16625762	28.02%
**Mapping statistics of sample CTX24_1**					
Total Reads	59113242	100.00%	Total Reads	59113242	100.00%
Total BasePairs	5320191780	100.00%	Total BasePairs	5320191780	100.00%
Total Mapped Reads	48370304	81.83%	Total Mapped Reads	47333514	80.07%
Perfect Match	38005859	64.29%	Perfect Match	39305533	66.49%
Mismatch	10364445	17.53%	Mismatch	8027981	13.58%
Unique Match	40975401	69.32%	Unique Match	44350708	75.03%
Multi-position Match	7394903	12.51%	Multi-position Match	2982806	5.05%
Total Unmapped Reads	10742938	18.17%	Total Unmapped Reads	11779726	19.93%
**Mapping statistics of sample CTX24_2**					
Total Reads	59367344	100.00%	Total Reads	59367344	100.00%
Total BasePairs	5343060960	100.00%	Total BasePairs	5343060960	100.00%
Total Mapped Reads	48891411	82.35%	Total Mapped Reads	44448516	74.87%
Perfect Match	40039761	67.44%	Perfect Match	38093562	64.17%
Mismatch	8851650	14.91%	Mismatch	6354954	10.70%
Unique Match	42533356	71.64%	Unique Match	41890560	70.56%
Multi-position Match	6358055	10.71%	Multi-position Match	2557956	4.31%
Total Unmapped Reads	10475933	17.65%	Total Unmapped Reads	14918826	25.13%
**Mapping statistics of sample CTX84_1**					
Total Reads	58744940	100.00%	Total Reads	58744940	100.00%
Total BasePairs	5287044600	100.00%	Total BasePairs	5287044600	100.00%
Total Mapped Reads	48343901	82.29%	Total Mapped Reads	46633980	79.38%
Perfect Match	36827585	62.69%	Perfect Match	37483892	63.81%
Mismatch	11516316	19.60%	Mismatch	9150088	15.58%
Unique Match	40595732	69.11%	Unique Match	43597780	74.22%
Multi-position Match	7748169	13.19%	Multi-position Match	3036200	5.17%

Total Unmapped Reads	10401039	17.71%	Total Unmapped Reads	12110958	20.62%
**Mapping statistics of sample CTX84_2**					
Total Reads	61019836	100.00%	Total Reads	61019836	100.00%
Total BasePairs	5491785240	100.00%	Total BasePairs	5491785240	100.00%
Total Mapped Reads	50114789	82.13%	Total Mapped Reads	49279020	80.76%
Perfect Match	38267912	62.71%	Perfect Match	39737307	65.12%
Mismatch	11846877	19.41%	Mismatch	9541713	15.64%
Unique Match	43477604	71.25%	Unique Match	46322134	75.91%
Multi-position Match	6637185	10.88%	Multi-position Match	2956886	4.85%
Total Unmapped Reads	10905047	17.87%	Total Unmapped Reads	11740814	19.24%

Supplementary 5. Primer information of the genes in real-time fluorescent qPCR.

Gene description	Forward primer	Reverse primer
Pax7	TCTCCAAGATTCTGTGCCGAT	CGGGGTTCTCTCTCTTATACTCC
Myod1	CCACTCCGGGACATAGACTTG	AAAAGCGCAGGTCTGGTGAG
MyH3	AAAAGGCCATCACTGACGC	CAGCTCTCTGATCCGTGTCTC
Ccl2	TTAAAAACCTGGATCGGAACCAA	GCATTAGCTTCAGATTTACGGGT
Ccl3	TTCTCTGTACCATGACACTCTGC	CGTGGAATCTTCCGGCTGTAG
Ccl4	TTCCTGCTGTTTCTCTTACACCT	CTGTCTGCCTCTTTTGGTCAG
Tnfa	CCCTCACACTCAGATCATCTTCT	GCTACGACGTGGGCTACAG
IL6	TAGTCCTTCCTACCCCAATTTCC	TTGGTCCTTAGCCACTCCTTC
IL1b	GCAACTGTTCCTGAACTCAACT	ATCTTTTGGGGTCCGTCAACT
Myf5	CCTGTCTGGTCCCGAAAGAAC	GACGTGATCCGATCCACAATG
Myogenin	GAGACATCCCCCTATTTCTACCA	GCTCAGTCCGCTCATAGCC

Supplementary 6. Fold change of 41 candidate immune factors expression level between healthy muscle and injured muscle.

Fold change of 41 candidate immune factors expression level in injured muscle compared to NC.

Gene ID	Symbol	Fold
20202	S100a9	117.3712
12986	Csf3r	52.54554
330122	Cxcl3	42.01269
57349	Ppbp	37.14154
20302	Ccl3	33.62097
16181	Il1rn	31.71117
17474	Clec4d	27.57464
16176	Il1b	26.05024
