# Supplementary material for: Transcriptome analysis of injured muscle identifies new candidate genes for satellite cell growth and myofiber formation during early muscle regeneration
Source: Anim Biosci. 2025 Aug 12;39(2):240859. doi: 10.5713/ab.24.0859 (PMC12877386; doi:10.5713/ab.24.0859)
Supplement: Supplementary file 5 [file ab-24-0859-Supplementary-5.pdf]

Total Unmapped Reads	10401039	17.71%	Total Unmapped Reads	12110958	20.62%
**Mapping statistics of sample CTX84_2**					
Total Reads	61019836	100.00%	Total Reads	61019836	100.00%
Total BasePairs	5491785240	100.00%	Total BasePairs	5491785240	100.00%
Total Mapped Reads	50114789	82.13%	Total Mapped Reads	49279020	80.76%
Perfect Match	38267912	62.71%	Perfect Match	39737307	65.12%
Mismatch	11846877	19.41%	Mismatch	9541713	15.64%
Unique Match	43477604	71.25%	Unique Match	46322134	75.91%
Multi-position Match	6637185	10.88%	Multi-position Match	2956886	4.85%
Total Unmapped Reads	10905047	17.87%	Total Unmapped Reads	11740814	19.24%

Supplementary 5. Primer information of the genes in real-time fluorescent qPCR.

Gene description	Forward primer	Reverse primer
Pax7	TCTCCAAGATTCTGTGCCGAT	CGGGGTTCTCTCTCTTATACTCC
Myod1	CCACTCCGGGACATAGACTTG	AAAAGCGCAGGTCTGGTGAG
MyH3	AAAAGGCCATCACTGACGC	CAGCTCTCTGATCCGTGTCTC
Ccl2	TTAAAAACCTGGATCGGAACCAA	GCATTAGCTTCAGATTTACGGGT
Ccl3	TTCTCTGTACCATGACACTCTGC	CGTGGAATCTTCCGGCTGTAG
Ccl4	TTCCTGCTGTTTCTCTTACACCT	CTGTCTGCCTCTTTTGGTCAG
Tnfa	CCCTCACACTCAGATCATCTTCT	GCTACGACGTGGGCTACAG
IL6	TAGTCCTTCCTACCCCAATTTCC	TTGGTCCTTAGCCACTCCTTC
IL1b	GCAACTGTTCCTGAACTCAACT	ATCTTTTGGGGTCCGTCAACT
Myf5	CCTGTCTGGTCCCGAAAGAAC	GACGTGATCCGATCCACAATG
Myogenin	GAGACATCCCCCTATTTCTACCA	GCTCAGTCCGCTCATAGCC

Supplementary 6. Fold change of 41 candidate immune factors expression level between healthy muscle and injured muscle.

Fold change of 41 candidate immune factors expression level in injured muscle compared to NC.

Gene ID	Symbol	Fold
20202	S100a9	117.3712
12986	Csf3r	52.54554
330122	Cxcl3	42.01269
57349	Ppbp	37.14154
20302	Ccl3	33.62097
16181	Il1rn	31.71117
17474	Clec4d	27.57464
16176	Il1b	26.05024
